# *Notes from the Field:* Measles Outbreaks from Imported Cases in Orthodox Jewish Communities — New York and New Jersey, 2018–2019

**DOI:** 10.15585/mmwr.mm6819a4

**Published:** 2019-05-17

**Authors:** Robert McDonald, Patricia Schnabel Ruppert, Maria Souto, Dylan E. Johns, Kevin McKay, Noelle Bessette, Lissette X. McNulty, Jennifer E. Crawford, Patrick Bryant, Maria Cecilia Mosquera, Sonya Frontin, Tatiana Deluna-Evans, Daniel E. Regenye, Elizabeth F. Zaremski, Vanessa J. Landis, Bonnie Sullivan, Brian E. Rumpf, Judi Doherty, Kathryn Sen, Eric Adler, Lisa DiFedele, Stephanie Ostrowski, Christine Compton, Elizabeth Rausch-Phung, Irina Gelman, Barbara Montana, Debra Blog, Bradley J. Hutton, Howard A. Zucker

**Affiliations:** ^1^Epidemic Intelligence Service, CDC; ^2^New York State Department of Health; ^3^Rockland County Department of Health, New York; ^4^New Jersey Department of Health; ^5^Orange County Department of Health, New York; ^6^Ocean County Health Department, Toms River, New Jersey; ^7^New York State Department of Health, Wadsworth Center; ^8^Metropolitan Area Regional Office, New York State Department of Health.

On October 1, 2018, the Rockland County (New York) Department of Health (RCDOH) alerted the New York State Department of Health (NYSDOH) of an unvaccinated teenaged traveler with diagnosed measles. During the next 17 days, RCDOH learned of an additional six unvaccinated travelers with measles. On October 24, 2018, the Ocean County (New Jersey) Health Department alerted the New Jersey Department of Health (NJDOH) of a case of measles in an international traveler, with rash onset October 17. The unvaccinated travelers reported recent travel in Israel, where an outbreak of approximately 3,150 cases of measles is ongoing (*1*). Investigations during October 1, 2018–April 30, 2019, identified 242 laboratory-confirmed and epidemiologically linked measles cases in New York, excluding New York City, and during October 17, 2018–November 30, 2018, identified 33 in New Jersey (Figure). The cases of measles were primarily in members of orthodox Jewish communities.

**FIGURE:**
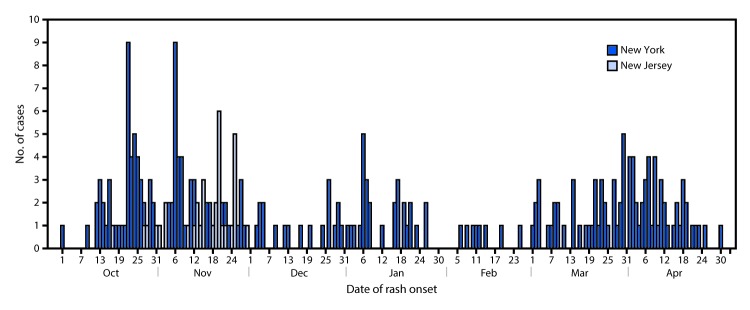
Number of measles cases, by date of rash onset — New York (n = 242)* October 1, 2018–April 30, 2019, and New Jersey (n = 33) October 17, 2018–November 30, 2018 * Excludes New York City.

## New York

The 242 cases in New York (excluding New York City) included 206 in Rockland County and 36 in nearby counties. Most patients resided in orthodox Jewish neighborhoods with low school immunization rates. The median patient age was 5 years (range = 0 days* to 63 years). The 2017–2018 New York State School Immunization Survey measles vaccination rate for students in prekindergarten through grade 12 was 98%; however, documented measles vaccination coverage in schools in the outbreak area was only 77%. To prevent disease spread in schools, Rockland County and neighboring Orange County have excluded unvaccinated students from school for 21 days after a measles exposure. To further control spread after school exposures, in areas of Rockland County with measles cases, exclusions from school were expanded to include all nonimmune students at schools that had measles immunity rates of <95% as documented by 2 valid doses of measles-mumps-rubella vaccine (MMR) or serologic evidence of immunity. To provide opportunities for vaccination, approximately 20 community vaccination events open to all ages were held in Rockland County and two in Orange County.

During October 1, 2018–April 30, 2019, Rockland County administered 19,661 MMR doses. NYSDOH, RCDOH, and private medical providers held nine informational events and distributed educational materials focused on measles prevention to 45,000 homes. A culturally appropriate and detailed vaccine education book was distributed to 15,000 Rockland County and 10,000 Orange County homes and medical providers. Orthodox Jewish leaders were engaged in the outbreak response, with rabbinical leaders supporting vaccination efforts and community groups advocating for vaccination. As of April 30, 2019, transmission was ongoing. This has been the largest measles outbreak in New York (outside New York City) since 1992 and, at 7 months, the longest documented outbreak in the United States since endemic measles was eliminated in 2000 (*2*).

## New Jersey

During October 17–November 30, 2018, 33 measles cases were confirmed in New Jersey, primarily in members of the orthodox Jewish community in Ocean County. The median patient age was 10 years (range = 6 months–59 years). In Ocean County, unvaccinated students were excluded from school for 21 days after a measles exposure. Some private schools excluded unvaccinated students for the duration of the New Jersey outbreak. NJDOH worked with local health officials and providers to facilitate delivery of >12,500 outbreak response doses of MMR vaccine to Ocean County medical providers. This outbreak was declared over on January 16, 2019. A second outbreak occurred in the same community in March 2019, with no identified connection to the first outbreak.

In the New York outbreak, low community vaccination rates facilitated widespread measles transmission after introduction of imported measles in unvaccinated travelers. Educational efforts regarding risks associated with undervaccination should be increased in communities with low vaccination rates. Health departments and clinicians should be aware of multiple ongoing measles outbreaks globally, and travelers should have evidence of measles immunity.^†^ All U.S. communities should maintain ≥95% levels of age-appropriate vaccination coverage with 2 doses of MMR vaccine to ensure herd immunity (*3*).

## Footnotes

* A newborn infant tested positive for measles following birth to a mother who had measles during pregnancy.

† Documentation of age-appropriate vaccination with a live measles virus-containing vaccine (1 dose for infants aged 6–11 months; 2 doses for persons aged ≥12 months, with the first dose administered at age ≥12 months and the second dose ≥28 days after the first dose); or laboratory evidence of immunity; or laboratory confirmation of measles disease; or birth before 1957.
